# Structural basis for sequence-specific DNA recognition by a group IId WRKY transcription factor GhWRKY17 in cotton

**DOI:** 10.1042/BCJ20250191

**Published:** 2026-01-22

**Authors:** Qin Xiao, Yu Wang, Xinci Shang, Yichang Chen, Ming Zhang, Yinhao Zhou, Xiaolei Huang, Su Qin, Jinrong Min, Guoqiang Xu, Yanli Liu

**Affiliations:** 1 Jiangsu Key Laboratory of Drug Discovery and Translational Research for Brain Diseases, College of Pharmaceutical Sciences, Soochow University, Suzhou, Jiangsu, 215123, China; 2 Jiangsu Province Engineering Research Center of Precision Diagnostics and Therapeutics Development, Soochow University, Suzhou, Jiangsu, 215123, China; 3 Suzhou International Joint Laboratory for Diagnosis and Treatment of Brain Diseases, Suzhou, Jiangsu, 215123, China; 4 Laboratory of Pesticides and Chemistry Key Laboratory of Ministry of Education, School of Life Sciences, Central China Normal University, Wuhan, Hubei, 430079, China; 5 Life Science Research Center, Southern University of Science and Technology, Shenzhen, Guangdong, 518055, China; 6 Structural Genomics Consortium and Department of Physiology, University of Toronto, Toronto, Ontario, M5G, 1L7, Canada

**Keywords:** GhWRKY17 WRKY domian, HOX3 W-box dsDNA, complex structure

## Abstract

WRKY transcription factors, a plant-specific family of transcriptional regulators, are classified into four groups (I–IV) and play pivotal roles in plant defense, development, and stress responses. These proteins are characterized by conserved WRKY domains that preferentially bind to the W-box cis-element C/TTGACC/T in target gene promoters. In *Gossypium hirsutum* (Gh; upland cotton), the group IId member GhWRKY17 regulates cotton fiber development by activating downstream target genes such as *GhHOX3* through promoter W-box binding. However, the structural basis for its DNA recognition specificity remains elusive. Here, we present the 1.8 Å resolution crystal structure of the GhWRKY17 WRKY domain in complex with the *GhHOX3* promoter DNA—the first structural characterization of a group IId WRKY protein. Structural analysis reveals that it consists of four antiparallel β-strands, with the β2-strand (harboring the conserved ^249^WRKYGQK^255^ motif) and β3-strand co-operatively engaging the DNA major groove. Key residues (R250, K251, Y252, Q254, K255, R264, Y266, Y267) form an intricate hydrogen-bonding network essential for recognizing the extended G/TTTGACC motif. Comparative structural analyses with group I/IIa/III WRKY–DNA complexes reveal that GhWRKY17’s dual-strand engagement and extensively hydrogen bond-mediated specific interaction represent novel mechanistic features distinguishing group IId members from other WRKY subgroups, emphasizing the necessity for subgroup-specific investigations. These findings not only establish a structural paradigm for group IId WRKY function but also provide molecular insights for engineering cotton fiber traits through transcriptional regulation.

## Introduction

Transcription factors are crucial regulators of plant growth and development [1–3]. The expression of many genes in plants, as well as their responses to specific stimuli, depends on the interaction between transcription factors and corresponding cis-acting elements [4–6]. This interaction can activate or inhibit gene transcription, thereby co-ordinating cross-talk between signaling pathways [2,5,7,8]. Among these regulators, WRKY proteins represent one of the largest plant-specific families, orchestrating diverse processes from stress responses to organ morphogenesis [7,8]. The WRKY transcription factors have been extensively studied in various plants, including *Arabidopsis thaliana* (At; mouse-ear cress) [9], *Oryza sativa* (Os; rice) [10], *Gossypium hirsutum* (Gh; upland cotton) [11], and *Glycine max* (Gm; soybean) [12], where they function distinctly in different plant tissues and developmental stages [7,8].

WRKY transcription factors are defined by the presence of one or more WRKY domains, a DNA-binding domain composed of ∼60 amino acid residues that preferentially binds to the DNA sequence C/TTGACC/T, termed W-box, with a universally conserved core TGAC sequence [8,13,14]. These domains are characterized by a conserved WRKYGQK motif at the N-terminus and a zinc-finger-like motif at the C-terminus, with two types: a zinc‑finger motif with two cysteines and two histidines (C_2_-H_2_; C-X_4-5_-C-X_22-23_-H-X-H) or C_2_-HC (C-X_7_-C-X_23_-H-X-C) [13,15]. Based on the number of WRKY domains and the type of zinc-finger-like motifs, WRKY transcription factors are divided into four groups (I–IV) [14,15]. Group I: dual WRKY domains (N- and C-terminal) with C_2_-H_2_ zinc-finger-like motifs. While the C-terminal WRKY domain (C-WRKY) mediates primary DNA binding, the N-terminal WRKY domain (N-WRKY) enhances affinity through co-operative interactions [16,17]. However, recent structural studies revealed unexpected DNA-binding capacity in AtWRKY1’s N-WRKY domain [18]. Group II: single WRKY domain with C_2_-H_2_ zinc-finger-like motif [8,14,15], subdivided into six subgroups (IIa–IIf) by sequence phylogeny [8,19]. Group III: single WRKY domain with distinct C_2_-HC zinc-finger-like motif, categorized into IIIa/IIIb [8,15,19,20]. Group IV: truncated WRKYGQK motif lacking zinc co-ordination [20]. Despite structural elucidation of group I (AtWRKY4; Protein Data Bank (PDB) 2LEX) [21] and group III (OsWRKY45; PDB 6IR8) [22] complexes, group II WRKYs—particularly the agriculturally vital IId subgroup—remain structurally enigmatic.

In *G. hirsutum*, GhWRKY17 (alternatively GhWRKY16) emerges as a group IId transcriptional activator critical for fiber initiation and elongation [23,24]. It drives developmental programs by binding W-box elements in promoters of master regulators like *GhHOX3*, *GhMYB109,* and *GhMYB25* [24–27]. Notably, GhHOX3 promotes the elongation of upland cotton fibers [26]. Meanwhile, MYB transcription factors GhMYB109 and GhMYB25 are crucial for fiber growth and fiber cell differentiation [24,25,27]. Therefore, by binding to W-box cis-acting elements in the promoters of these target genes, GhWRKY17 activates their transcription, thus facilitating both fiber initiation and elongation [23]. However, the structural determinants governing its DNA recognition specificity remain unresolved. By combining X-ray crystallography, biophysical profiling, and functional mutagenesis, we decrypt the molecular logic underlying GhWRKY17’s DNA recognition—revealing both conserved principles and subgroup-specific innovations that redefine our understanding of WRKY function.

## Results and discussion

### GhWRKY17 WRKY domain binds to W-Box sequences in target promoters

**Figure 1:**
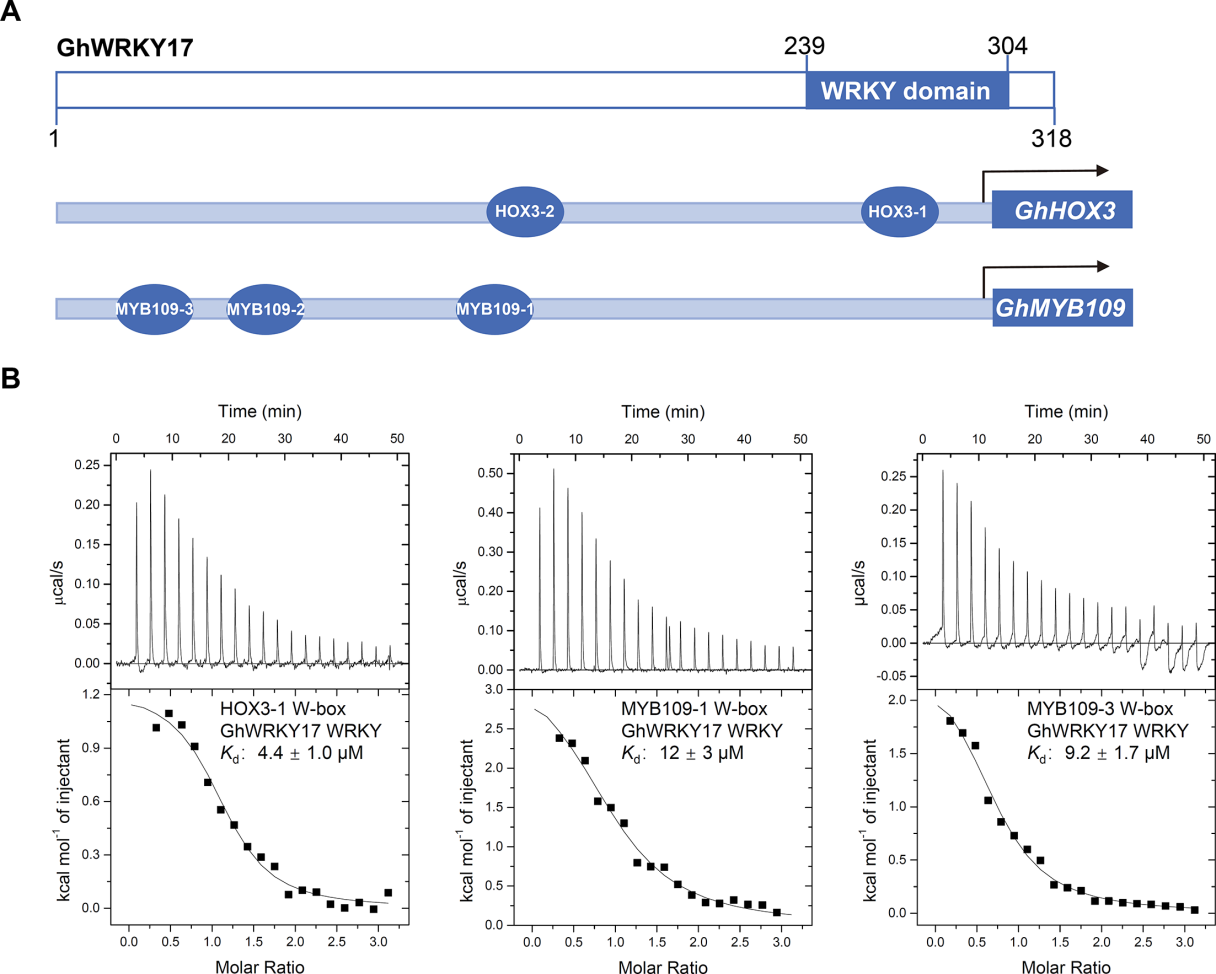
DNA-binding ability of GhWRKY17 WRKY domain. (**A**) Domain architecture of GhWRKY17 and schematic of W-box elements in *GhHOX3* and *GhMYB109* promoters. (**B**) Isothermal titration calorimetry (ITC) curves for GhWRKY17 WRKY domain (residues 239–304) binding to 12 bp W-box duplexes (HOX3-1, MYB109-1, and MYB109-3). Dissociation constants (*K*_d_, μM) were calculated from heat differentials after buffer control subtraction. Data represent two independent replicates (MicroCal iTC-200).

Previous studies established that GhWRKY17 binds to W-box sequences in the promoters of target genes such as *GhHOX3* and *GhMYB109* to regulate cotton fiber development [23] (Figure 1A, Supplementary Table S1). To confirm that this interaction is mediated by the WRKY domain of GhWRKY17 directly, we performed an isothermal titration calorimetry (ITC) assay with purified recombinant GhWRKY17 WRKY domain (residues 239–304) and three synthetic 12 bp W-box duplexes (HOX3-1, MYB109-1, and MYB109-3, Figure 1A). ITC measurements revealed micromolar-range binding affinities across all tested sequences, with dissociation constants (*K*_d_) ranging from 4.4 to 12 μM (Figure 1B). These comparable affinities demonstrate the WRKY domain’s intrinsic capacity for W-box recognition, independent of full-length protein context.

Comparative analysis revealed evolutionary tuning of DNA-binding energetics that GhWRKY17’s affinity parallels group IIa AtWRKY18 (5–6 μM) [28] and group III OsWRKY45 (4.6 μM) [22], but is markedly weaker than group I AtWRKY1 (0.1 μM) [18]. This subgroup-dependent affinity hierarchy suggests functional specialization, where group I WRKYs may require stronger DNA binding for rapid response, while group II members like GhWRKY17 employ moderate affinity suited for developmental regulation.

### Crystal structure of GhWRKY17 WRKY–dsDNA complex

**Figure 2:**
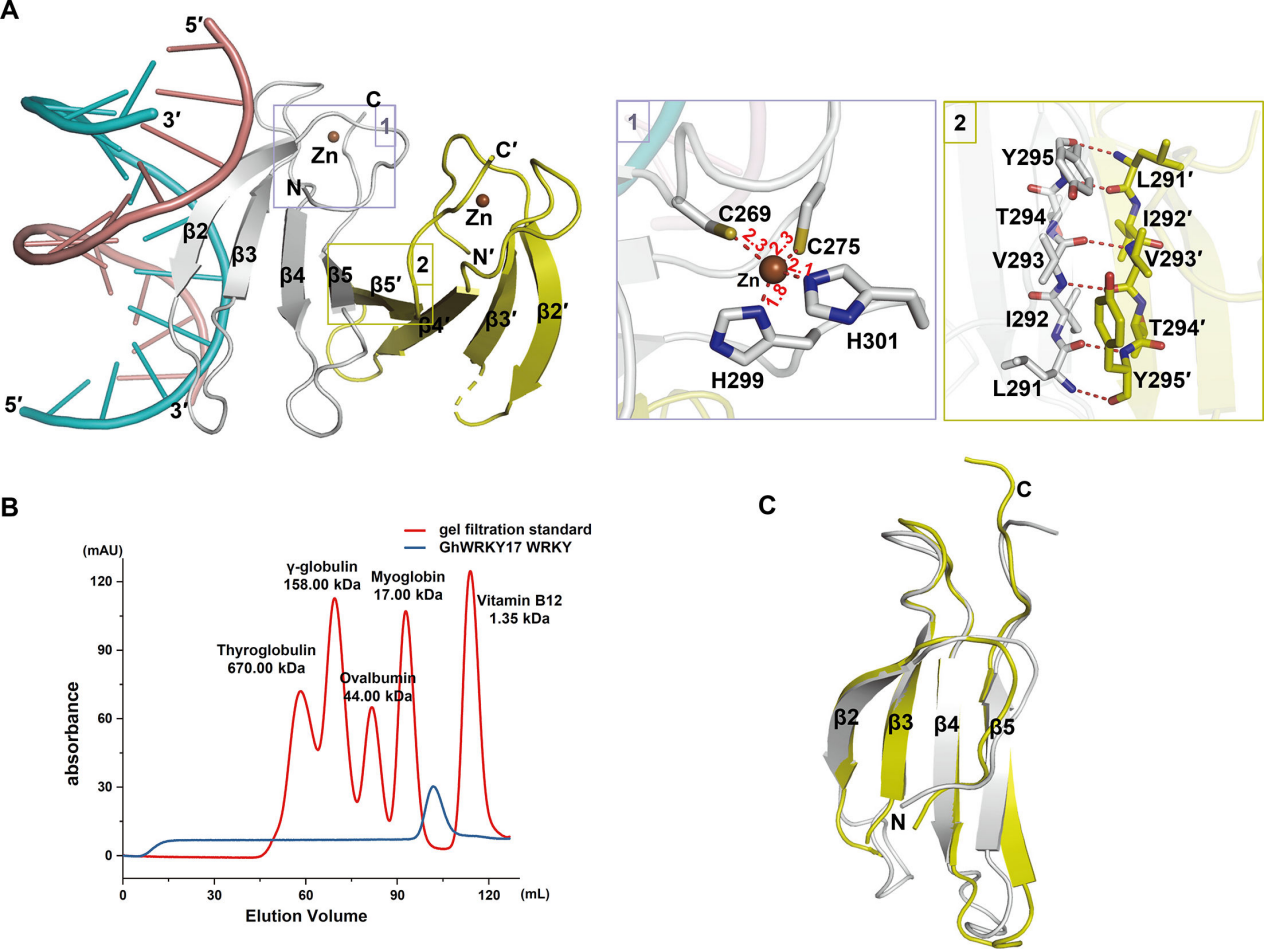
Crystal structure of GhWRKY17 WRKY in complex with HOX3-1 W-box dsDNA. (**A**) Crystal structure of GhWRKY17 WRKY dimer (gray/yellow) bound to W-box dsDNA (sense strand: salmon; antisense strand: cyan). Zinc ions (brown spheres) co-ordinate with conserved residues. Insets: zinc-binding site (left) and dimer interface (right). (**B**) Size-exclusion chromatography (SEC) profile (Superdex™ 75 pg) showing monomeric state of GhWRKY17 WRKY domain in solution (blue) versus protein standards (red, Bio-Rad). (**C**) Structural superposition of DNA-bound (gray) and DNA-free (yellow) WRKY domains.

**Figure 3:**
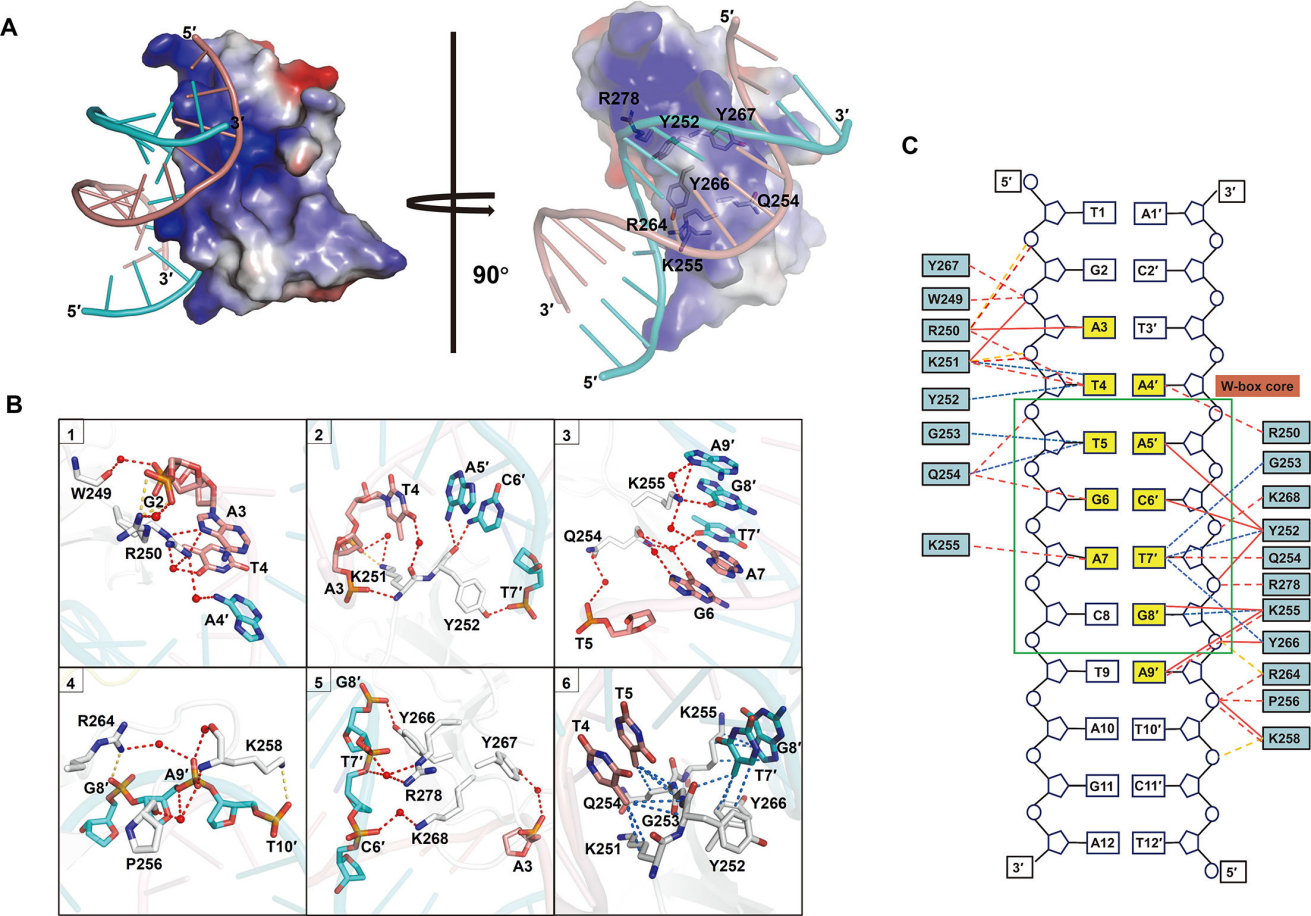
Interaction network of GhWRKY17 WRKY and HOX3-1 W-box dsDNA. (**A**) Electrostatic surface potential of the GhWRKY17 WRKY–DNA complex (left) and rotated 90° view (right), highlighting DNA-binding cleft. (**B**) Detailed interactions between GhWRKY17 WRKY domain and HOX3-1 W-box dsDNA. The key residues of the WRKY domain and nucleotides of sense and antisense strands were shown as gray, salmon, and cyan sticks, respectively. Key hydrogen bonds, electrostatic interactions, and hydrophobic contacts were shown as red, orange, and blue dashed lines, respectively. Key water molecules were depicted as red spheres. (**C**) Schematic view of the interactions between the GhWRKY17 WRKY domain and HOX3-1 W-box dsDNA. Bases specifically recognized by the WRKY domain were highlighted in yellow, the core TGAC motif was highlighted by a green box, and binding involved direct and water-mediated hydrogen bonds, electrostatic interactions, and hydrophobic contacts were shown as solid red, dashed red, dashed orange, and dashed blue lines, respectively.

To elucidate the molecular mechanism of DNA recognition by the transcription factor GhWRKY17, we attempted to crystallize its WRKY domain in complex with three W-box double-stranded DNA (dsDNA) sequences (HOX3-1, MYB109-1, and MYB109-2). Notably, we successfully determined the crystal structure of the GhWRKY17 WRKY domain bound to a 12 bp HOX3-1 W-box dsDNA at 1.8 Å resolution—the first reported complex structure for a group IId WRKY transcription factor and the first structure-solved WRKY transcription factor in *G. hirsutum* (Figures 2–3, Table 1).

**Table 1:** Data collection and refinement statistics

	GhWRKY17 WRKY–*GhHOX3* dsDNA
**PDB code**	9M0K
**Data collection**	
Space group	C222_1_
Cell dimensions	
*a*, *b*, *c* (Å)	54.1, 93.3, 83.7
*α*, *β*, *γ* (◦)	90, 90, 90
Resolution (Å)	46.81~1.80 (1.84~1.80)
Measured reflections	37,707 (2085)
Unique reflections	19,996 (1105)
*R*_merge_ (%)	0.039 (0.219)
*I*/*σI*	20.4 (4.4)
CC_1/2_	0.997 (0.895)
Completeness (%)	99.7 (96.2)
Redundancy	1.9 (1.9)
**Refinement**	
Resolution (Å)	46.81~1.80 (1.86~1.80)
*R*_work_/*R*_free_ (%)	24.6/27.9
No. of atoms/average *B*-factors (Å^2^)	1650/34.1
Protein	982/32.5
Ions	2/34.6
Ligand	486/35.5
Water	180/39.3
Root mean square deviation	
Bond lengths (Å)	0.01
Bond angles (°)	1.35
Ramachandran plot % residues	
Favored	100.00

Values in parentheses are for the highest resolution shell.

The asymmetric unit contains two WRKY monomers and one DNA duplex, with only one monomer engaging in direct DNA binding (Figure 2A). Each monomer adopts a canonical WRKY fold, consisting of four antiparallel β-strands: β2 (W249–K255; to maintain consistency with previously published structures, the β-strand bearing the conserved WRKY signature motif was named as β2), β3 (R264–C269), β4 (R278–A284), and β5 (M290–E296). A C_2_-H_2_ zinc-finger-like motif stabilizes the structure, co-ordinated by C269 and C275 (β3–β4 loop) and H299/H301 (C-terminal loop), with zinc-ligand distances within ideal ranges: 2.3 Å (Zn–C269), 2.3 Å (Zn–C275), 1.8 Å (Zn–H299), and 2.1 Å (Zn–H301) (Fig. 2A-1) [29]. Intriguingly, the two WRKY monomers form a homodimer via antiparallel β5–β5′ interactions, stabilized by hydrogen bonds and hydrophobic contacts among residues ^291^LIVTY^295^ (Figure 2A–2). However, size-exclusion chromatography (SEC) revealed a monomeric state in solution (Figure 2B), suggesting dimerization is either crystallization-induced or concentration-dependent—a phenomenon consistent with observations in AtWRKY18 [28] and other previous studies [30,31]. Notably, superposition of DNA-bound and free WRKY domains revealed minimal conformational changes (root mean square deviations ∼0.9 Å; Figure 2C), indicating a rigid-body binding mode without significant structural rearrangement upon DNA interaction.

### Atomic-level interaction network between GhWRKY17 WRKY domain and W-box dsDNA

The crystal structure reveals a canonical WRKY–dsDNA binding geometry, with the β-sheet (β2–β5) orthogonally inserted into the DNA major groove (Figure 2A). Electrostatic surface analysis identified a positively charged cleft of the GhWRKY17 WRKY domain facilitating DNA engagement (Figure 3A). For systematic interaction mapping, we designated positions 1–12 (5′→3′) on the sense strand and 12′–1′ (5′→3′) on the antisense strand, with the core TGAC motif spanning positions 5–8 (sense) and 8′–5′ (antisense) (Figure 3B–C). The key interactions include the conserved WRKYGQK motif driving core recognition and the extended interaction network stabilizing the complex.

**Figure 4:**
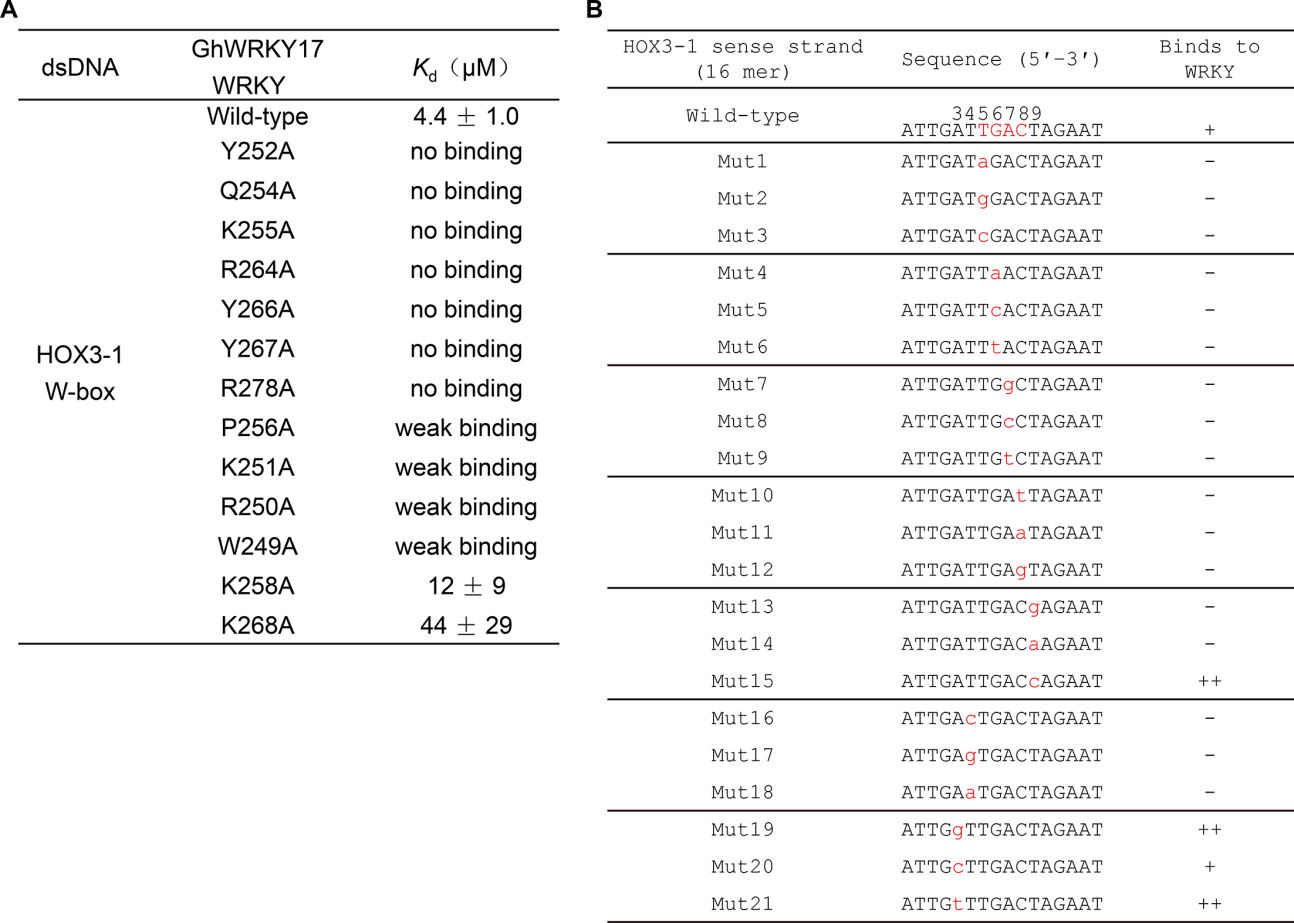
Mutagenesis reveals critical binding determinants. (**A**) Dissociation constants (*K*_d_, μM) of HOX3-1 W-box dsDNA to wildtype and mutant GhWRKY17 WRKY domains determined by ITC. Shown are representative of two independent experiments. (**B**) Binding ability of 16 bp wildtype (core TGAC in red) and mutant HOX3-1 W-box dsDNA (mutated bases in red lowercase) to GhWRKY17 WRKY domain determined by electrophoretic mobility shift assays (EMSA). Only the sequence of the sense strand was shown with the serial number above the wildtype sequence kept consistent with the 12-mer DNA sequence used in the complex structure for clear understanding.

**Figure 5:**
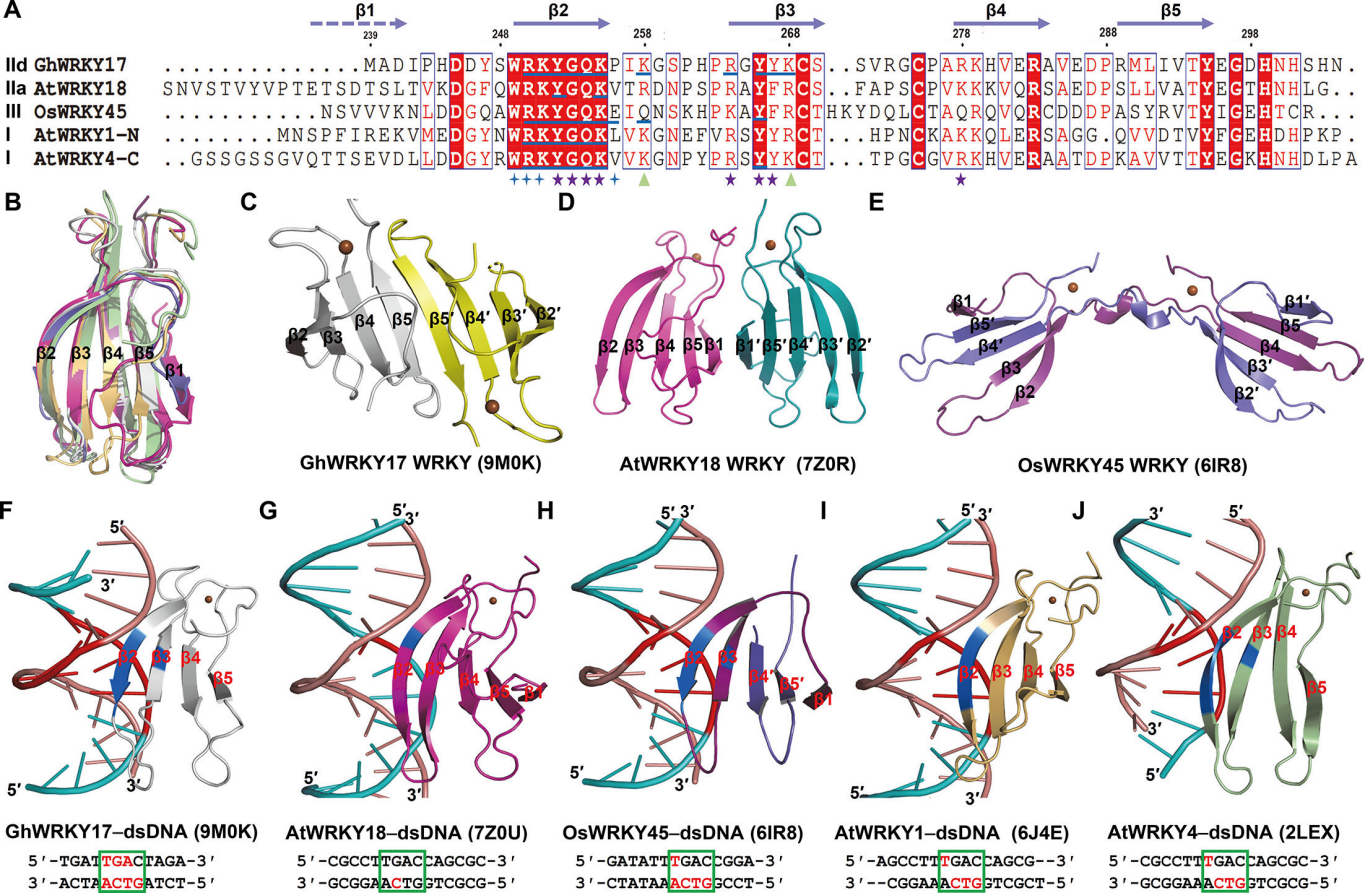
Divergence of WRKY–DNA interactions. (**A**) Structure-guided sequence alignment of selected WRKY domains (group IId GhWRKY17, group IIa AtWRKY18, group I AtWRKY1 N-WRKY, group I AtWRKY4 C-WRKY, and group III OsWRKY45). Key DNA interaction residues of GhWRKY17 WRKY were shown as purple★ (essential), blue ✦ (necessary), and green ▲ (auxiliary), respectively. The residues involved in specific base recognition were marked with blue underlines. Missing β1 strand in GhWRKY17 WRKY was indicated by a dashed arrow. (**B**) Structural superposition of selected WRKY domains, GhWRKY17 (gray), AtWRKY18 (pink), AtWRKY1 N-WRKY (golden), AtWRKY4 C-WRKY (green), and OsWRKY45 (violet and deep purple). (**C–E**) Dimerization interfaces of GhWRKY17 (β5–β5′, **C**), AtWRKY18 (β1–β1′, **D**), and OsWRKY45 (β4–β5 swap + Zn²^+^, **E**). (**F–J**) Base specific recognition of the TGAC motif by different WRKY domains. The specific interaction involved residues and nucleotides are colored in blue and red, respectively, and the core TGAC motif was highlighted by a green box. Gh, *Gossypium hirsutum* (upland cotton); At, *Arabidopsis thaliana* (mouse-ear cress); Os, *Oryza sativa* (rice).

The signature ^249^WRKYGQK^255^ motif on β2 mediates critical DNA contacts. (1) W249 anchors the complex via water-bridged hydrogen bonds between its backbone carbonyl and A3 phosphate (Figure 3B-1, 3C, and Supplementary Table S2). (2) R250 exhibits dual conformational states: state 1, the guanidinium group forms several direct/water-mediated hydrogen bonds with A3 (purine), T4 (pyrimidine), and A4′ (purine); and state 2, the guanidinium group interacts with the G2 phosphate via direct/water-mediated hydrogen bonds and electrostatic interactions (Figure 3B-1, 3C, and Supplementary Table S2). (3) K251 engages T4 phosphate through its side chain, while backbone groups contact A3 phosphate and T4 pyrimidine (Figure 3B-1, 3C, and Supplementary Table S2). (4) Y252 hydroxyl directly binds to T7′ phosphate, with the main chain carbonyl recognizing amine groups of A5′ and C6′ (Figure 3B-1, 3C, and Supplementary Table S2). (5) Q254 co-ordinates T5 phosphate via its side chain, while backbone interactions stabilize G6 and T7′ (Figure 3B–3C, and Supplementary Table S2). (6) K255 bridges G8′, A9′, and A7 through multipoint recognition (Figure 3B–3C, and Supplementary Table S2). In addition to these major interactions, the side chains of K258/R264/Y266/Y267/K268/R278 and the main chains of P256/K258 form hydrogen bonds and electrostatic interactions with A3 and C6′–T10′ phosphates, enhancing binding ability (Figures 3B–4, B–5 and 3C, and Supplementary Table S2). Furthermore, hydrophobic contacts between ^251^KYGQK^255^/Y266 and T4/T5/T7′/G8′ bases further enforce sequence selectivity (Figures 3B–6, 3C, and Supplementary Table S2), consistent with WRKY family mechanisms [18,21,22,28].

In summary, the interaction landscape identifies three co-operative elements that the β2 strand (^250^RKYGQK^255^) orchestrates core TGAC recognition, β3 strand (R264/Y266/Y267/K268) stabilizes flanking regions, and adjacent residues (K258/R278) fine-tune phosphate contacts. This multivalent binding strategy explains GhWRKY17’s specificity for the bipartite ^3^ATTGACT^9^ motif across both DNA strands. The integration of conformational plasticity (R250), water-mediated bridging, and modular domain contributions exemplifies evolutionary optimization for developmental gene regulation.

### Mutagenesis reveals critical binding determinants

To identify the key residues involved in DNA binding, we generated point mutations in the GhWRKY17 WRKY domain and quantitatively assessed their binding affinities using ITC. Strikingly, alanine substitutions at residues Y252, Q254, K255, R264, Y266, Y267, and R278 completely disrupt DNA binding (Figure 4A; Supplementary Figure S1), highlighting their indispensable role in the interaction. Structural analyses revealed that these residues directly engage with the W-box core sequence through side-chain interactions, where mutations disrupt critical hydrogen bonds, electrostatic interactions, and hydrophobic contacts with DNA nucleotides (Figure 3B–C). Furthermore, mutations of P256, K251, R250, and W249 to alanine nearly eliminate the DNA binding (Figure 4A; Supplementary Figure S1), underscoring their substantial contribution to binding stability (Figure 3). In contrast, substitutions at K258 and K268 only moderately reduce binding affinity (3 and 10 times weaker compared with the wildtype, Figure 4A; Supplementary Figure S1), suggesting their auxiliary roles in the interaction.

Notably, our findings align with prior mutagenesis studies of analogous residues in other WRKY domains, with a few exceptions [18,32,33]. For example, while the Q254A mutation in GhWRKY17 (corresponding to Q121A in AtWRKY1 N-WRKY) abrogates DNA binding, the equivalent Q317A mutation in AtWRKY1 C-WRKY retains binding activity [18,33]. These discrepancies highlight both conserved and divergent functional features among WRKY domains, emphasizing the necessity for subgroup-specific investigations.

### Sequence-specific recognition of the W-box

To investigate the functional significance of specific nucleotides within the W-box sequence, we performed electrophoretic mobility shift assays (EMSA) using synthetic 16 bp wildtype or mutant DNAs and the wildtype GhWRKY17 WRKY domain (Supplementary Table S1). Consistent with our structural data, mutations in the core TGAC sequence (Mut1–Mut12) completely disrupt DNA binding (Figure 4B; Supplementary Figure S2A), confirming the indispensability of this motif, as previously reported for group IIb (AtWRKY6) and IId (AtWRKY11) WRKY proteins [32]. Intriguingly, this contrasts with group IIc (AtWRKY43), group I (AtWRKY26), and group III (AtWRKY38) proteins, which exhibit divergent nucleotide preferences [32].

To dissect flanking nucleotide roles, we analyzed mutations adjacent to the core TGAC sequence (Figure 4B; Supplementary Figure S2B). (1) Position T9 (3′ to TGAC): Mut13/14 (T9→G/A, corresponding to A9′→C/T) abolishes binding due to disrupted hydrogen bonds between K255 and the purine ring of A9′ (Figure 3B–C; Supplementary Figure S3A). Mut15 (T9→C, corresponding to A9′→G) enhances binding (Figure 4B; Supplementary Figure S2B), likely due to structural similarities between adenine and guanine and additional hydrogen bonds mediated by the carbonyl group of guanine with the side chain of K255 (Figure 3B–C; Supplementary Figure S3A). These data indicate that the GhWRKY17 WRKY domain prefers a base C to T at the position 9, the 3′ adjacent to the core TGAC sequence. This finding contrasts with AtWRKY6/AtWRKY11 (preferring T significantly) and AtWRKY43 (requiring C absolutely) [32], but is comparable to AtWRKY18 (no C/T bias) [28].

(2) Position T4 (5′ to TGAC): Mut16–18 (T4→C/G/A) eliminates binding (Figure 4B; Supplementary Figure S2B), likely due to disrupted hydrogen bonds (side chain of R250 with T4’s pyrimidine ring) and hydrophobic interactions (K251/Y252 with T4’s methyl group) (Figure 3B–C; Supplementary Figure S3B), highlighting T4 as another critical nucleotide for the specific interaction. This mirrors AtWRKY6/AtWRKY11 but opposes AtWRKY43/AtWRKY26/AtWRKY38, which favor T→G/A substitutions [32].

(3) Position A3: Mut19/21 (A3→G/T) enhances binding (Figure 4B; Supplementary Figure S2B), suggesting that A3 plays an auxiliary role in stabilizing interactions and the GhWRKY17 WRKY domain prefers G or T at this site (Figure 3B–C; Supplementary Figure S3C). This diverges from AtWRKY6/AtWRKY11 (G-specific) but resembles AtWRKY43/AtWRKY26/AtWRKY38 (no significant nucleotide preference) [32].

Collectively, our data reveal that GhWRKY17 WRKY preferentially binds to the W-box sequence G/TTTGACC, with distinct nucleotide requirements at flanking positions compared with other WRKY subgroups. These findings underscore the functional divergence among WRKY transcription factors and emphasize the necessity for subgroup-specific structural studies to elucidate their DNA recognition mechanisms.

### Structural and functional divergence among WRKY subgroups

The GhWRKY17 WRKY–*GhHOX3* DNA complex structure represents two major progresses—the first resolved WRKY transcription factor complex in *G. hirsutum* (upland cotton) and the inaugural structural characterization of a group IId WRKY–DNA interaction. Currently, six WRKY–DNA complexes are available in the PDB, including four group I structures (AtWRKY4 C-WRKY, AtWRKY1 N-WRKY, AtWRKY2 N-WRKY, and AtWRKY33 N-WRKY), one group IIa (AtWRKY18), and one group III (OsWRKY45) [18,21,22,28]. Sequence and structural analyses revealed both conserved and divergent features among WRKY domains. Sequence alignment demonstrated that the amino acid residues mediating interactions between the GhWRKY17 WRKY domain and *GhHOX3* DNA are conserved across other WRKY domains (Figure 5A). Structural superimposition further confirmed this conservation, despite variations in β-sheet composition (four or five antiparallel β strands), with the signature WRKYGQK motif consistently positioned on the outermost β2 strand (Figure 5B).

Notably, the crystal structure of the GhWRKY17 WRKY domain complexed with *GhHOX3* DNA reveals a dimeric configuration (Figure 2A). Comparative analysis with existing structures shows that while group IIa (AtWRKY18) and group III (OsWRKY45) WRKY transcription factors also exhibit dimeric states, group I members do not (Figure 5C–E) [18,22,28]. However, the dimerization mechanism varies among subgroups. GhWRKY17 forms dimers through hydrogen bonds between β5 and β5′ strands (Figure 5C), while AtWRKY18 utilizes β1 and β1′ interactions (Figure 5D) [28]. In contrast, OsWRKY45 exhibits a unique domain-swapping stabilized by zinc ions (Figure 5E) [22]. SEC analyses revealed that while GhWRKY17 and AtWRKY18 WRKY domains exist as monomers in solution, OsWRKY45 maintains an oligomeric state. This dynamic oligomerization implies weaker interdomain interactions in group II WRKYs compared with the zinc-stabilized quaternary structure of group III members.

DNA recognition analyses across WRKY subgroups revealed both conserved and divergent features. All studied WRKY domains employ a β-sheet insertion into the DNA major groove, with the β2 strand (bearing the WRKYGQK motif) universally engaging the TGAC core (Figure 5F–J) [18,21,22,28]. Structural comparisons highlight the crucial role of conserved tyrosine (Y) and the second lysine (K) in the heptapeptide sequence for W-box recognition (Figure 5A). However, further investigation uncovered that GhWRKY17 WRKY exhibits two distinctive features. It recognizes the TGAC motif on both sense and antisense strands, in contrast to other WRKYs that primarily interact with the TGAC complementary sequence on the antisense strand (Figure 5F–J, specific interaction involved nucleotides highlighted in red), and its DNA binding predominantly relies on hydrogen bonding, differing from the extensive hydrophobic interactions observed in other subgroups (Figure 3, Supplementary Figure S4), [18,21,22,28]. Notably, the specific multipoint recognition of bases G6 and A7 represents a novel feature first reported in the GhWRKY17–DNA complex (Figure 5F–J, Supplementary Figure S4). Mutagenesis studies corroborated these structural observations, showing differential contributions of conserved residues and nucleotides to WRKY–DNA interaction across subgroups (Figure 4) [18,21,22,28,32–34].

These findings highlight an evolutionary paradox. While WRKY domains maintain a conserved DNA-binding scaffold, subgroup-specific variations in oligomerization strategies, secondary structure utilization, and interaction chemistries (hydrogen bonds *vs.* hydrophobic interactions) suggest functional specialization. Such structural plasticity likely enables fine-tuning of DNA recognition mechanisms to fulfill distinct regulatory roles in plant development and stress responses.

## Conclusions

In this study, we have deciphered the molecular basis of DNA recognition by the WRKY domain of GhWRKY17, establishing the first structural framework for group IId WRKY transcription factors. Through integrated structural and mutagenesis analyses, we demonstrate that GhWRKY17 exhibits unique sequence specificity, preferentially binding to the G/TTTGACC motif through selective recognition of nucleotides flanking the core TGAC element. This discovery extends previous functional observations showing GhWRKY17’s critical role in cotton fiber development via promoter W-box interactions [23]. Comparative analyses with other WRKY–DNA complexes reveal an intriguing evolutionary paradigm. While all WRKY domains share a conserved β-sheet DNA-binding scaffold, subgroup-specific variations in sequence recognition strategies and interaction chemistries (e.g. hydrogen bonding *vs.* hydrophobic contacts) underscore functional diversification. Notably, GhWRKY17’s dual-strand engagement and extensively hydrogen bond-mediated specific interaction represent novel mechanistic features distinguishing group IId members from other WRKY subgroups. These findings not only advance our understanding of cotton fiber morphogenesis at the molecular level but also provide a structural blueprint for precision breeding strategies. The identified DNA-binding specificity could guide targeted promoter engineering to optimize GhWRKY17-regulated pathways, potentially enhancing fiber yield and quality. Future studies should address the structural basis of WRKY subgroup specialization through high-resolution characterization of diverse family members, which may uncover universal principles governing plant transcriptional regulation.

## Materials and methods

### Protein expression and purification

The coding sequence for the GhWRKY17 WRKY domain (residues 239–304) was cloned into a pET28-MHL vector (Addgene; cat. 26096) to generate an N-terminal 6 × His-TEV-tagged construct using seamless assembly cloning (ABclonal Technology; cat. RK21020). Sequence-verified plasmids (Azenta Life Sciences) were transformed into *E. coli* BL21(DE3) Codon Plus RIL cells (TransGen; cat. CD601). Protein expression was induced with 0.25 mM IPTG at OD_600_=0.8, followed by incubation at 15°C for 24 h. Cells were lysed in buffer (20 mM Tris-HCl pH 7.5, 250 mM NaCl, 5% glycerol, 5 mM β-mercaptoethanol), and the lysate was purified via Ni-nitrilotriacetate (Ni-NTA) affinity chromatography (GE Healthcare; cat. 17526802) using stepwise imidazole gradients (wash: 40 mM; elution: 250 mM). The His-tag was cleaved by TEV protease during dialysis in 20 mM Tris-HCl pH 7.5 and 150 mM NaCl, and tag-free protein was further purified by SEC (Superdex™ 75 pg, GE Healthcare; cat. 28989335) in 20 mM Tris-HCl pH 7.5, 150 mM NaCl, and 1 mM DTT and concentrated using Amicon Ultra-15 Centrifugal Filter Units (Millipore Corporation; cat. UFC901024). Site-directed mutants were generated using the Fast Mutagenesis System (TransGen; cat. FM111-02), with sequences confirmed by Sanger sequencing (Azenta Life Sciences). Mutant proteins were overexpressed and purified as the wildtype protein described above.

### DNA preparation

Synthetic oligonucleotides containing W-box sequences from *GhHOX3* (HOX3-1) and *GhMYB109* (MYB109-1, MYB109-3) promoters (Synbio Technologies) were annealed in 20 mM Tris-HCl pH 7.5, 150 mM NaCl by heating to 95°C (3 min) followed by gradual cooling to 4°C.

### Isothermal titration calorimetry (ITC)

ITC experiments were performed on an iTC-200 microcalorimeter (Malvern Panalytical) at 25°C. The concentrated GhWRKY17 WRKY proteins and W-box dsDNAs were diluted into 20 mM Tris-HCl pH 7.5, 150 mM NaCl (ITC buffer). GhWRKY17 WRKY (50 μM in the cell chamber) was titrated with 750 μM 12 bp dsDNA (in the syringe) in 20 successive injections with a spacing of 150 s. Control experiments were performed under identical conditions to determine the heat signals that arise from injection of the dsDNA into the buffer (buffer dilution heats). Data were corrected for buffer dilution heats and analyzed using a single-site binding model in Origin 7.0 (MicroCal).

### Crystallization and structure determination

The GhWRKY17 WRKY: HOX3-1 W-box dsDNA complex (1:1.2 molar ratio) was crystallized at 18°C via sitting-drop vapor diffusion (0.5 μl protein/DNA mixture + 0.5 μl reservoir: 20% PEG 3350, 0.2 M ammonium formate) with a protein concentration of 6 mg/ml. X-ray diffraction data were collected at Canadian Light Source (CLS) 08ID and Shanghai Synchrotron Radiation Facility (SSRF) BL10U2, BL02U1 (λ=0.978 Å, 100 K). Diffraction images were processed using autoPROC [35] and XDS [36]. The structure was solved by molecular replacement with program PHASER [37] and co-ordinates from PDB entry 2AYD (apo structure of AtWRKY1-C WRKY domain) [33]. The DNA model was built manually in Coot [38] by fitting ideal B-form DNA fragments into the clearly defined electron density, followed by iterative refinement of base pairing and backbone geometry. The complex structure was further refined with REFMAC [39], PHENIX [40], and validated with MOLPROBITY [41].

### Electrophoretic mobility shift assay (EMSA)

Binding reactions (15 μl: 32 μM WRKY domain, 8 μM 16 bp dsDNA in 10 mM Tris-HCl pH 7.5, 50 mM NaCl, 1 mM EDTA, 10% glycerol, 1 mM DTT) were incubated on ice (30 min), resolved on 6% native polyacrylamide gels in 0.5 × Tris-Borate-EDTA (TBE) buffer at 100 V for 90 min, and visualized with SYBR™ Gold (Thermo Fisher; cat. S11494).

### Size-exclusion chromatography (SEC) analysis

SEC was performed on a Superdex™ 75 column (GE Healthcare; cat. 28989335) pre-calibrated with gel filtration standards (Bio-Rad; cat. 1511901) in 20 mM Tris-HCl pH 7.5, 150 mM NaCl, and 1 mM DTT.

## Supplementary material

online supplementary figure 1

## Data Availability

The atomic co-ordinates and structure factors have been deposited in the PDB (accession code: 9M0K [42]). Additional data are available in the Supplementary Information or from the authors upon request.

## Open Access

This article has been published open access under our Subscribe to Open programme, made possible through the support of our subscribing institutions, learn more here: https://portlandpress.com/pages/open_access_options_and_prices#conditional

## Abbreviations

At: *Arabidopsis thaliana* (mouse-ear cress)
C_2_-H_2_: zinc‑finger motif with two cysteines and two histidines
C-WRKY: C-terminal WRKY domain
EMSA: electrophoretic mobility shift assays
Gh: *Gossypium hirsutum* (upland cotton)
ITC: isothermal titration calorimetry
N-WRKY: N-terminal WRKY domain
Os: *Oryza sativa* (rice)
PDB: Protein Data Bank
SEC: size-exclusion chromatography
TEV: tobacco etch virus
dsDNA: double-stranded DNA
