# Supplementary material for: Structural basis for sequence-specific DNA recognition by a group IId WRKY transcription factor GhWRKY17 in cotton
Source: Biochem J. 2026 Jan 22;483(2):149–60. doi: 10.1042/BCJ20250191 (PMC12905487; doi:10.1042/BCJ20250191)
Supplement: online supplementary figure 1 [file bcj-483-2-BCJ20250191-s001.pdf]

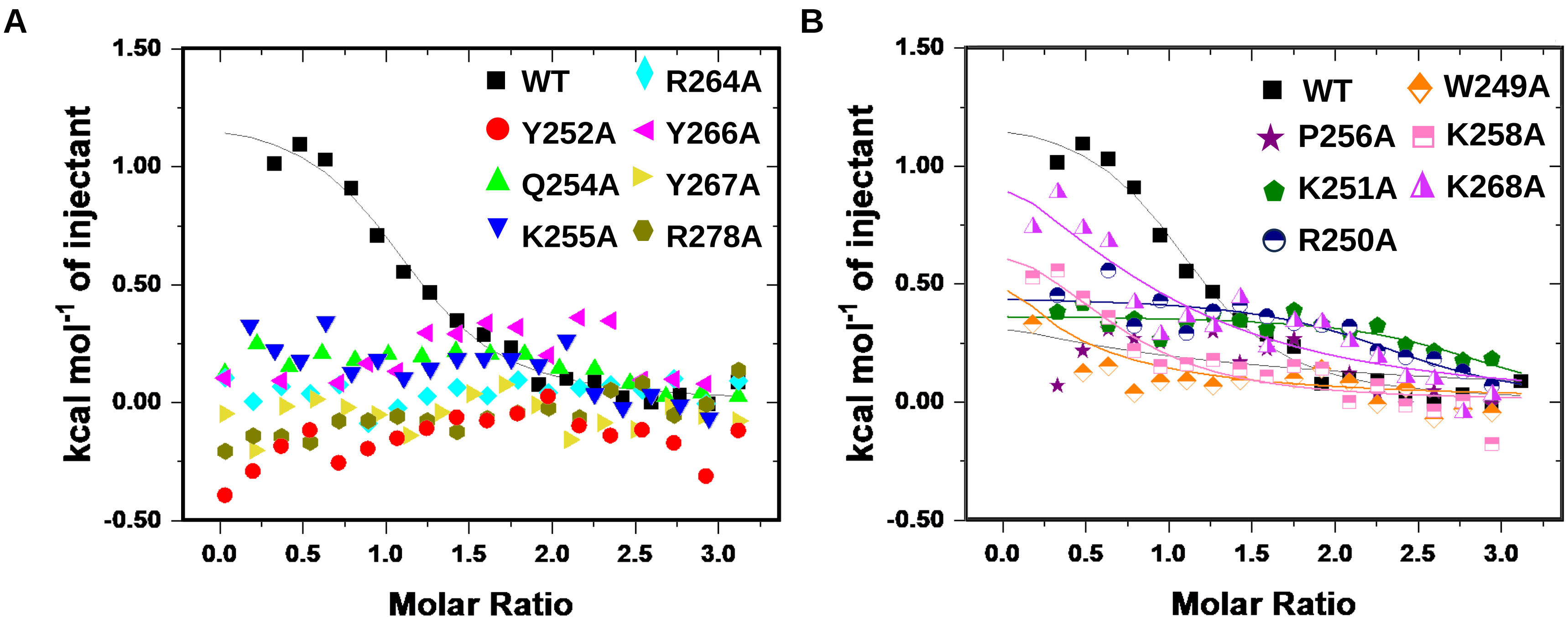

Supplementary Figure S1. ITC binding data for wild-type and mutant WRKY domains binding to HOX3-1 W-box dsDNA. **(A)** Disruption of DNA binding by essential residue mutations. **(B)** Reduced affinity with auxiliary residue mutations.

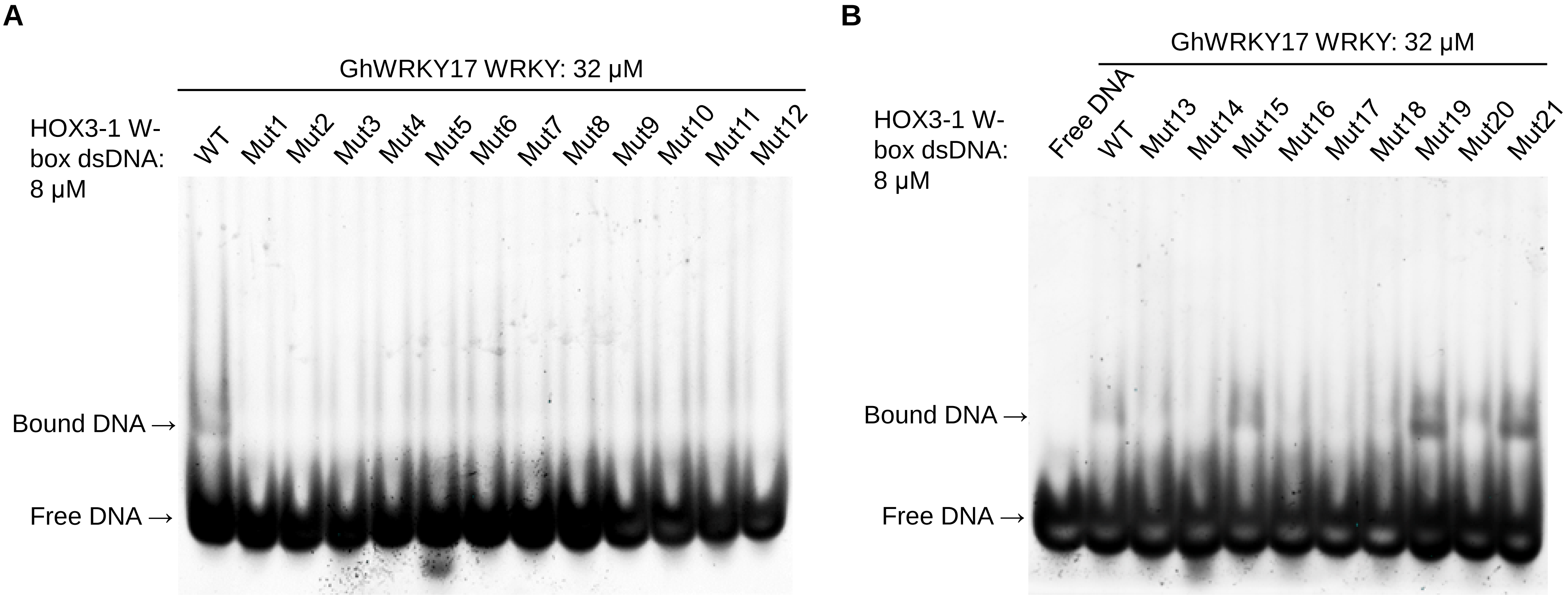

Supplementary Figure S2. Sequence specificity of W-box recognition. (A–B) Electrophoretic mobility shift assays (EMSA) results of GhWRKY17 WRKY domain binding to wild-type (WT) and mutant W-box dsDNA. The core mutations **(A)** abolish binding, while flanking mutations **(B)** variably affect the interaction.

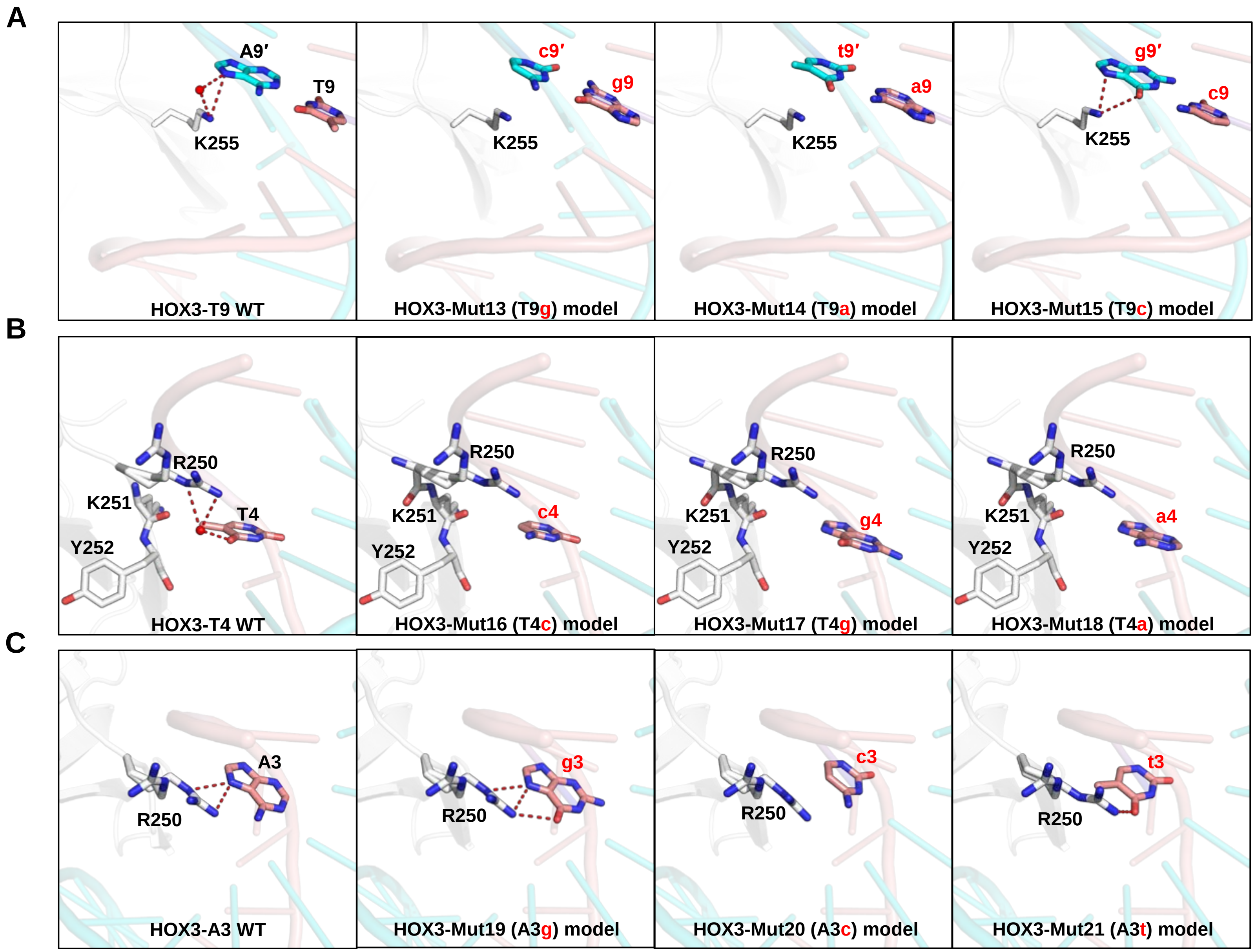

Supplementary Figure S3. Structure models of different GhHOX3-1 W-box dsDNA mutants bind to GhWRKY17 WRKY domain. **(A)** Models of T9 position mutants. **(B)** Models of T4 position mutants. **(C)** Models of A3 position mutants.

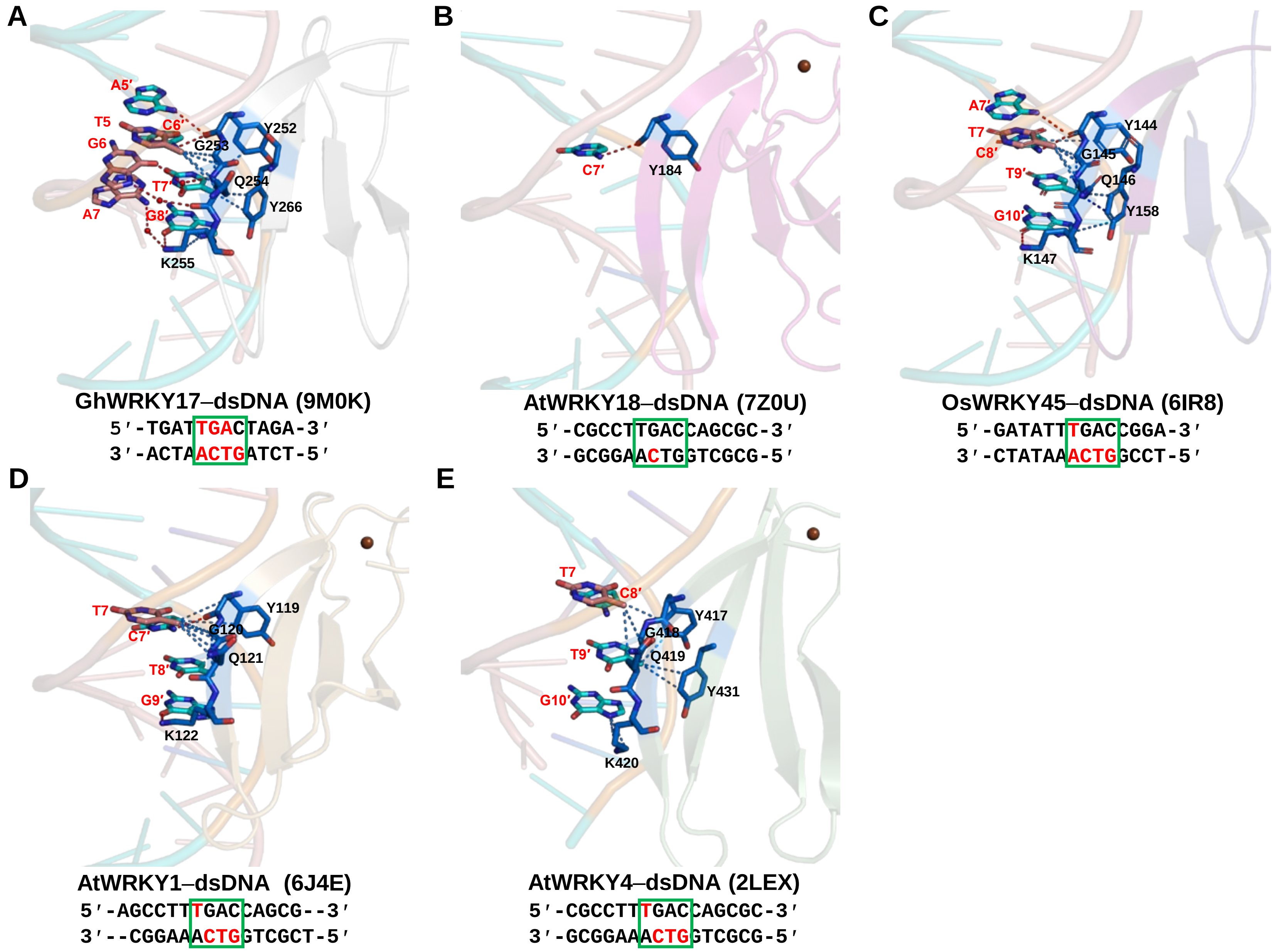

Supplementary Figure S4. Base specific recognition of TGAC motif by different WRKY domains. (A-E) GhWRKY17 (A), AtWRKY18 (B), OsWRKY45 (C), AtWRKY1 (D), AtWRKY4 (E), respectively. Specific interaction involved residues and nucleotides were shown as sticks and the variable interaction chemistries were shown as red (hydrogen bonds) and blue (hydrophobic contacts) dashed lines, respectively.

Supplementary table 1 W-box-containing DNA sequences

W-box DNA name	W-box DNA sequence
HOX3-1_sense	5′-TGATTGACTAGA-3′
HOX3-1_antisense	5′-TCTAGTCAATCA-3′
HOX3-2_sense	5′-GAAAGTCAAGGA-3′
HOX3-2_antisense	5′-TCCTTGACTTTC-3′
MYB109-1_sense	5′-TTATTGACTTGT-3′
MYB109-1_antisense	5′-ACAAGTCAATAA-3′
MYB109-2_sense	5′-TTGTTGACCCAG-3′
MYB109-2_antisense	5′-CTGGGTCAACAA-3′
MYB109-3_sense	5′-ACAAGTCAATTG-3′
MYB109-3_antisense	5′-CAATTGACTTGT-3′
**HOX3-1 W-box DNA for EMSA**	**W-box DNA sequence**
Wild-type_sense	5′-ATTGATTGACTAGAAT-3′
Wild-type_antisense	5′-ATTCTAGTCAATCAAT-3′
Mut1_sense	5′-ATTGATaGACTAGAAT-3′
Mut1_antisense	5′-ATTCTAGTCtATCAAT-3′
Mut2_sense	5′-ATTGATgGACTAGAAT-3′
Mut2_antisense	5′-ATTCTAGTCcATCAAT-3′
Mut3_sense	5′-ATTGATcGACTAGAAT-3′
Mut3_antisense	5′-ATTCTAGTCgATCAAT-3′
Mut4_sense	5′-ATTGATTaACTAGAAT-3′
Mut4_antisense	5′-ATTCTAGTtAATCAAT-3′
Mut5_sense	5′-ATTGATTcACTAGAAT-3′
Mut5_antisense	5′-ATTCTAGTgAATCAAT-3′

Mut6_sense	5′-ATTGATTtACTAGAAT-3′
Mut6_antisense	5′-ATTCTAGTaAATCAAT-3′
Mut7_sense	5′-ATTGATTGgCTAGAAT-3′
Mut7_antisense	5′-ATTCTAGcCAATCAAT-3′
Mut8_sense	5′-ATTGATTGcCTAGAAT-3′
Mut8_antisense	5′-ATTCTAGgCAATCAAT-3′
Mut9_sense	5′-ATTGATTGtCTAGAAT-3′
Mut9_antisense	5′-ATTCTAGaCAATCAAT-3′
Mut10_sense	5′-ATTGATTGAtTAGAAT-3′
Mut10_antisense	5′-ATTCTAaTCAATCAAT-3′
Mut11_sense	5′-ATTGATTGAaTAGAAT-3′
Mut11_antisense	5′-ATTCTAtTCAATCAAT-3′
Mut12_sense	5′-ATTGATTGAgTAGAAT-3′
Mut12_antisense	5′-ATTCTAcTCAATCAAT-3′
Mut13_sense	5′-ATTGATTGACgAGAAT-3′
Mut13_antisense	5′-ATTCTcGTCAATCAAT-3′
Mut14_sense	5′-ATTGATTGACaAGAAT-3′
Mut14_antisense	5′-ATTCTtGTCAATCAAT-3′
Mut15_sense	5′-ATTGATTGACcAGAAT-3′
Mut15_antisense	5′-ATTCTgGTCAATCAAT-3′
Mut16_sense	5′-ATTGAcTGACTAGAAT-3′
Mut16_antisense	5′-ATTCTAGTCAgTCAAT-3′
Mut17_sense	5′-ATTGAgTGACTAGAAT-3′
Mut17_antisense	5′-ATTCTAGTCAcTCAAT-3′
Mut18_sense	5′-ATTGAaTGACTAGAAT-3′

Mut18_antisense	5′-ATTCTAGTCAtTCAAT-3′
Mut19_sense	5′-ATTGgTTGACTAGAAT-3′
Mut19_antisense	5′-ATTCTAGTCAAcCAAT-3′
Mut20_sense	5′-ATTGcTTGACTAGAAT-3′
Mut20_antisense	5′-ATTCTAGTCAAgCAAT-3′
Mut21_sense	5′-ATTGtTTGACTAGAAT-3′
Mut21_antisense	5′-ATTCTAGTCAAaCAAT-3′

Note: The mutated bases of DNA for the EMSA assays shown in red lowercase.

Supplementary table 2 Detailed interactions between GhWRKY17 WRKY domain and HOX3-1 W-box dsDNA.

	WRKY-residue	W-box nucleotide	Distance of H-bond	Electrostatic interaction	Hydrophobic packing
Base-specific interactions	R250-guanidinium	A3-purine ring	3.0 Å, 3.1 Å		
		T4-pyrimidine ring	2.6 Å, 3.0 Å via H_2O		
		A4′-purine ring	3.2 Å, 3.4 Å via H_2O		
	K251-backbone carbonyl	T4-pyrimidine ring	2.6 Å, 2.8 Å via H_2O		
	Y252-backbone carbonyl	A5′-purine ring	3.4 Å		
		C6′-pyrimidine ring	2.7 Å		
	Q254-backbone carbonyl	G6-purine ring	2.7 Å, 2.9 Å via H_2O		
	Q254-backbone amine	T7′-pyrimidine ring	2.7 Å, 2.9 Å via H_2O		
	K255-side chain amine	A7-purine ring	2.6 Å, 3.1 Å via H_2O		
		G8′-purine ring	2.8 Å		
		A9′-purine ring	2.8 Å, 2.8 Å via H_2O		
	K251-side chain carbon atoms	T4-methyl group			+
	Y252-backbone carbon atom	T4-methyl group			+
	Y252-benzene ring	T7′-pyrimidine ring			+
	G253-backbone carbon atom	T5-, T7′-methyl group			+
	Q254-side/backbone carbon atoms	T5-methyl group			+
	K255-side chain carbon atoms	G8′-purine ring			+
	Y266-benzene ring	T7′-methyl group			+
	W249-backbone carbonyl	A3-phosphate	2.7 Å, 2.8 Å via H_2O		
	R250-guanidinium	G2-phosphate	2.9 Å, 3.0 Å via H_2O	+	
	K251-side chain amine	T4-phosphate	2.9 Å, 3.3 Å via H_2O	+	
	K251-backbone amine	A3-phosphate	2.7 Å		

DNA-backbone contacts	Y252-hydroxyl	T7′-phosphate	2.5 Å	
	Q254-side chain amine	T5-phosphate	3.3 Å, 3.3 Å via H_2O	
	P256-backbone carbonyl	A9′-phosphate	2.8 Å, 2.8 Å via H_2O	
	K258-side chain amine	T10′-phosphate		+
	K258-backbone amine	A9′-phosphate	2.9 Å	
	K258-backbone carbonyl	A9′-phosphate	2.8 Å, 2.9 Å via H_2O	
	R264-guanidinium	G8′-phosphate		+
		A9′-phosphate	3.1 Å, 3.2 Å via H_2O	
	Y266-hydroxyl	G8′-phosphate	2.7 Å	
	Y267-hydroxyl	A3-phosphate	2.4 Å, 3.1 Å via H_2O	
	K268-side chain amine	C6′-phosphate	2.5 Å, 2.6 Å via H_2O	
	R278-guanidinium	T7′-phosphate	2.9 Å, 3.0 Å via H_2O	
